# Deciphering Structure‐Bubble‐Mass Transfer Relationships in Water Electrolysis Through Electrode Morphology Regulation

**DOI:** 10.1002/advs.77658

**Published:** 2026-09-13

**Authors:** Zhiqing Tang, Ziyun Zhang, Huicun Gu, Baoxin Wu, Kejun Yan, Jiahui Luo, Yuting Jiang, Cailin Xiao, Yu Xu, Lin Zeng

**Affiliations:** ^1^ Shenzhen Key Laboratory of Hydrogen Energy Department of Mechanical and Energy Engineering Southern University of Science and Technology Shenzhen People's Republic of China; ^2^ SUSTech Energy Institute For Carbon Neutrality Southern University of Science and Technology Shenzhen People's Republic of China; ^3^ Department of Mechanical Engineering The Hong Kong Polytechnic University Kowloon Hong Kong SAR People's Republic of China

**Keywords:** bubble dynamics, electrode morphology, mass transport, oxygen evolution reaction, water electrolysis

## Abstract

Unraveling the regulation of electrode local geometric curvature on bubble dynamics is essential to mitigate mass transport losses for the oxygen evolution reaction (OER). Conventional studies emphasize high surface area and porosity while neglecting local geometric curvature and connectivity. Here we identify local electrode curvature as a physically grounded descriptor. Positive curvature at sharp tips concentrates the electric field, raises local gas supersaturation, and reduces the three‐phase contact line pinning, which confines growing bubbles and enables their release at a small size. Flat or concave surfaces lack these effects and trap gas. To validate this, positive curvature (κ_+_) nanoneedles (NN), near‐zero curvature (κ_0_) nanosheets (NS), and negative curvature (κ_−_) microporous (MP) electrodes are developed. At 200 mA cm^−2^, in situ high‐speed visualization demonstrates that NN exhibits a departure diameter of 45 µm, alongside the minimal mass transport overpotential (158.52 mV). NS delivers moderate bubble behavior and an intermediate overpotential (248.04 mV), while MP suffers severe bubble pinning despite a moderate 60 µm bubble size, resulting in the highest mass transport overpotential (252.98 mV). This work verifies that open convex architectures govern bubble kinetics far beyond porosity, providing universal design guidelines for high‐performance OER electrocatalysts.

## Graphical abstract

Electrode morphology is shown to govern oxygen‐bubble dynamics and interfacial mass transfer during water electrolysis. Positive‐curvature nanoneedles promote rapid release of small bubbles, sustain electrolyte renewal, and minimize mass‐transport losses, whereas flat and concave architectures increasingly retain bubbles. Surface curvature therefore emerges as a practical descriptor for designing high rate gas‐evolving electrodes.

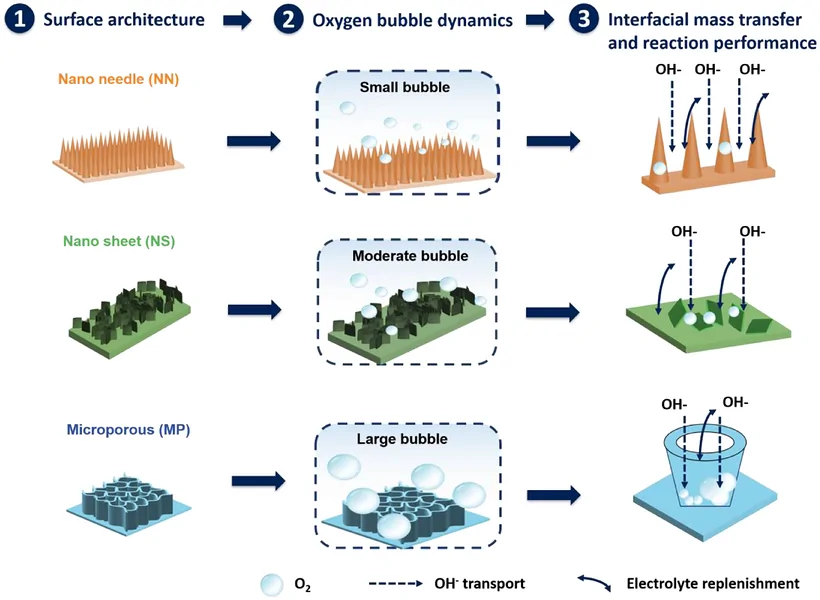

## 1 Introduction

Water electrolysis is one of the key technologies for producing green hydrogen, with strong potential in deep‐decarbonization applications such as ammonia synthesis, heavy‐duty transportation, steelmaking, and long‐duration energy storage. When powered by intermittent renewable electricity from wind or solar, electrolyzers enable scalable hydrogen production without direct greenhouse gas emissions. However, the high energy demand remains a major barrier to large scale commercialization [1, 2, 3].

During water electrolysis, H_2_ evolves at the cathode and O_2_ at the anode, generating microbubbles that nucleate, grow, coalesce, and detach on electrode surfaces [4, 5]. These bubbles are not merely passive by‐products; rather, they play an active role in shaping electrolyzer efficiency. Specifically, bubble accumulation decreases the effective electrochemically active area and hinders electrolyte renewal at the electrode‐electrolyte interface, thereby increasing ohmic and mass‐transport losses. As a result, a higher overpotential is required to sustain a given current density, leading to reduced overall energy efficiency and accelerated performance degradation. Therefore, promoting rapid bubble detachment and maintaining continuous interfacial renewal are crucial for efficient and durable water electrolysis.

Considerable progress has been made in improving the oxygen evolution reaction (OER), and existing strategies can generally be broadly categorized into two main directions. The first aims to enhance intrinsic catalytic activity through electronic‐structure engineering, for example by constructing heterostructures [6, 7, 8, 9], introducing dopants [10, 11, 12, 13], or creating vacancy defects [14, 15, 16, 17], thereby regulating the adsorption and conversion of key intermediates. However, this type of strategy mainly focuses on catalytic activity and fails to pay attention to the impact of bubble dynamics on performance. The second focuses on electrode morphology design, such as building 3D porous scaffolds to increase the electrochemically active surface area (ECSA) and expose more active sites, or introducing hierarchical pore networks to facilitate mass transport [18, 19, 20, 21]. For instance, Guo et al. developed a nickel‐foam‐supported 3D porous MXene‐based multi‐heterojunction transition‐metal phosphide electrode to enhance the apparent activity at high current densities [22]. Lv et al. reported vertically aligned porous feather‐like NiCoP nanoarrays, which improve electrolyte wetting through open channels and thereby accelerate the OER process [23]. Although these structural designs can increase ECSA and promote mass transport, they do not specifically address the problem of bubble accumulation, and most focus on a single morphology, making it impossible to distinguish the causal relationship between structure, bubble dynamics, and electrolytic performance.

Nevertheless, under vigorous gas evolution at high current densities, OER performance is not determined by ECSA and intrinsic activity alone, but also by bubble dynamics at the electrode surface. Bubble coverage, bubble coalescence, and bubble retention can impose substantial mass‐transfer penalties by limiting local electrolyte renewal near the interface and increasing interfacial mass‐transfer resistance, thereby reducing the effective utilization of active sites. In this regard, although nanosheet structures often provide a large ECSA, their characteristics may also promote bubble accumulation. As a result, the nominal surface‐area advantage does not necessarily translate into superior performance under high‐rate operating conditions.

To address these issues, a growing number of studies have begun to interpret the performance differences of gas‐evolving electrodes from the perspective of bubble dynamics. For example, Wang et al. discussed bubble coverage and detachment on CuOx/NiO nanosheet‐array electrodes and correlated these features with local interfacial mass transport and catalytic activity [24]. Lai et al. used Co_3_O_4_ nanoneedle arrays to construct superhydrophilic/superhydrophobic surfaces, thereby achieving weaker bubble adhesion and more facile bubble release [25]. Chu et al. investigated how the pore‐channel architecture and superhydrophilicity regulate bubble behavior within the channels of an ultrathin, nano‐honeycomb porous CoMoO_4_@pNi catalyst grown in situ on porous Ni (pNi) [26]. However, most existing studies remain largely qualitative and focus on a single morphology, while structural modulation is often accompanied by changes in composition and phase. This coupling makes it difficult to establish a causal relationship between electrode structure, bubble behavior, and electrochemical performance. Consequently, quantitative comparisons based on morphology‐controlled model systems remain scarce, and actionable design principles for bubble‐management‐oriented electrode architectures are still underdeveloped.

Motivated by this gap, we design and construct three classic model electrodes to systematically clarify how electrode morphology regulates bubble dynamics and further determines electrolysis performance. Numerous studies have shown that layered double hydroxides (LDHs) often form morphologies such as positive curvature (κ_+_) nanoneedles (NN), near‐zero curvature (κ_0_) nanosheets (NS), and negative curvature (κ_−_) microporous (MP); thus, we selected these morphologies to construct model electrodes. Three Cobalt‐Iron Layered Double Hydroxide (CoFe‐LDH) electrodes with well‐defined NN, NS, and MP morphologies were prepared with identical composition and surface chemistry to isolate the sole influence of morphology. High‐speed in situ imaging and FFT analysis were used to quantitatively investigate morphology‐dependent bubble dynamics.

Electrochemical measurements and MEA validation were further conducted to verify the relationship between bubble behavior and electrolysis performance. Finally, we propose rational design guidelines for high‐current‐density water electrolysis electrodes to enable efficient and durable operation.

## 2 Results and Discussion

### 2.1 Simulation

**FIGURE 1:**
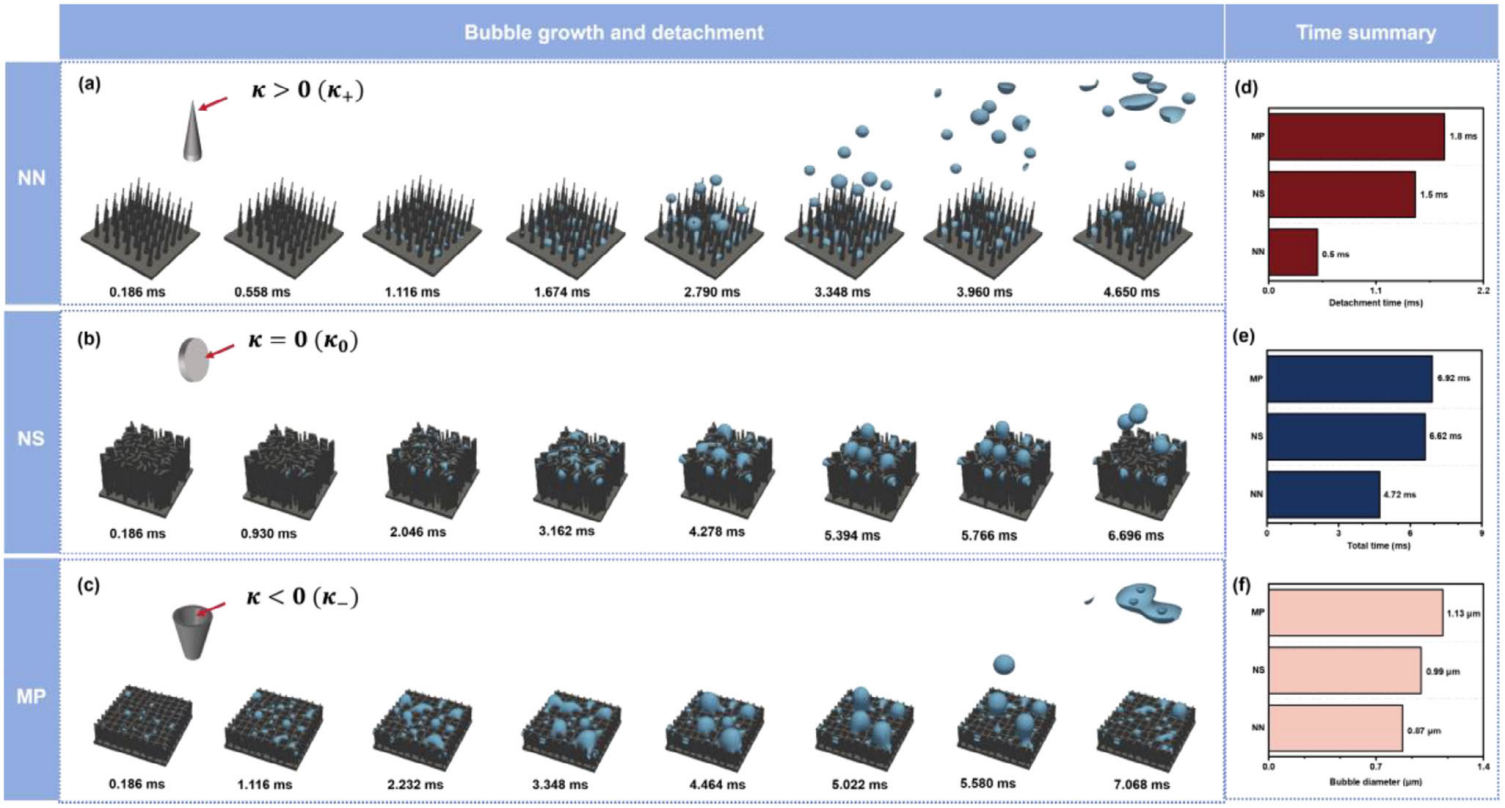
Evolution of the gas phase on the surface of NN (a), NS (b), and MP (c) morphologies, which involves two sequential stages of bubble growth and detachment. Comparison of bubble detachment (d) and total evolution (e) times across the three catalyst layer morphologies. (f) Simulated bubble detachment diameters of three catalyst morphologies.

To reveal the bubble evolution behavior on electrodes with different morphologies, a 3D two‐phase lattice Boltzmann (LB) model was used. Simulations were performed to compare bubble growth and detachment dynamics on three distinct catalyst layer morphologies, focusing on their temporal characteristics and bubble detachment diameter. Full details of the simulation method are given in the Supporting Information. Simulations revealed distinct bubble dynamics across the three morphologies (Figure 1). On the NN surface, bubbles detached earlier via local shedding and coalescence, exhibiting the shortest detachment duration (0.5 ms), the shortest total evolution time (4.72 ms), and the smallest average detachment diameter (0.87 µm). On the NS surface, bubbles grew and coalesced progressively, resulting in intermediate detachment duration (1.5 ms), total time (6.62 ms), and diameter (0.99 µm). On the MP surface, pore walls and intersection nodes enhanced bubble confinement, promoting retention and the formation of a dominant bubble before detachment, which gave the longest total evolution time (6.92 ms) and the largest detachment diameter (1.13 µm). These morphology‐governed trends predicted by the simulations were subsequently validated experimentally.

### 2.2 Morphology and Chemical Composition Analysis

**FIGURE 2:**
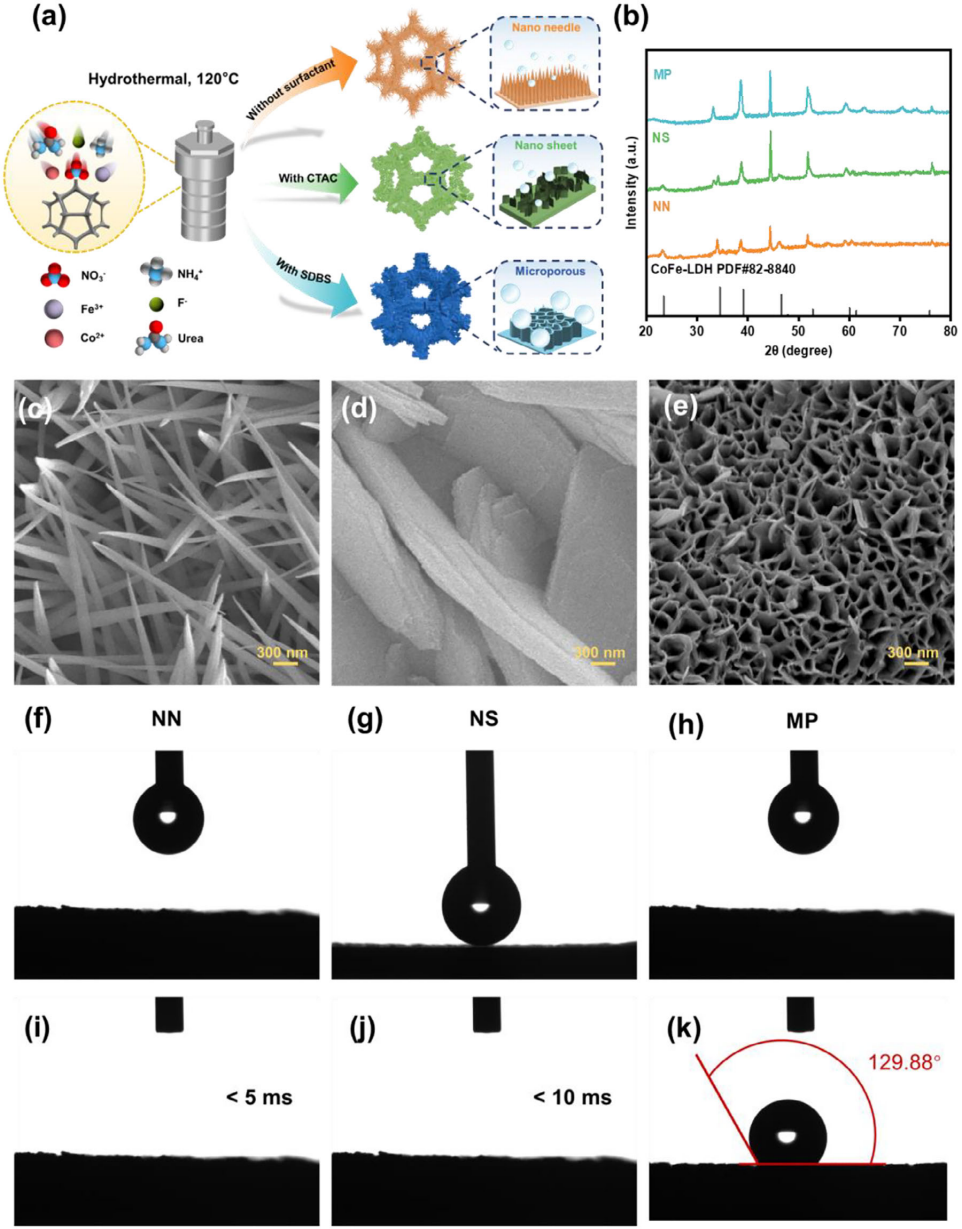
(a) Schematic illustration of the synthesis procedure for NN, NS, and MP. (b) XRD patterns of the NN, NS, and MP electrodes. SEM images of (c) NN, (d) NS, and (e) MP. Contact angles of (f, i) NN, (g, j) NS, and (h, k) MP.

Guided by the above simulation predictions, three CoFe‐LDH electrodes with targeted morphologies were rationally synthesized by only adjusting the surfactant, while all other synthetic parameters remained unchanged. The synthetic procedures of the three electrodes are illustrated in Figure 2a.

#### 2.2.1 Surface Morphology and Chemical Composition Analysis

Figure 2b shows the XRD patterns of the three CoFe‐LDH samples with different morphologies. All characteristic diffraction peaks match well with the standard CoFe‐LDH card (PDF#82‐8840). This indicates that all three electrodes are single phase CoFe‐LDH. The morphology control process changes only the morphology of the material, without introducing impurities or altering the crystal structure. X‐ray photoelectron spectroscopy (XPS) was used to analyze the elemental composition, chemical valence states, and surface electronic structure of the CoFe‐LDH catalysts to confirm that the NN, NS, and MP have the same chemical composition and differ only in morphology. As shown in Figure S1a, the survey spectra of the three samples are highly similar, showing characteristic peaks of Co, Fe, O, and C 1s (used for calibration), with no additional impurity signals detected. This preliminarily confirms that the three samples have the same surface elemental composition. The high‐resolution spectra of each element were further analyzed. The O 1s spectra (Figure S1b) of all three samples can be fitted with two components: the peak at 529.8 eV is assigned to metal‐oxygen bonds (M─O), and the peak at 531.2 eV corresponds to surface hydroxyl species (M─OH). The binding energies and relative intensities of these peaks are nearly identical among the three samples, indicating a similar chemical environment of surface oxygen species. For the active metal sites, the high‐resolution Co 2p spectra (Figure S1c) show a Co 2p_3/2_ main peak at 780.5 eV with obvious satellite peaks, which is characteristic of Co^3+^. In the high‐resolution Fe 2p spectra (Figure S1d), the Fe 2p_3/2_ main peak appears at 711.0 eV, and the peak shape and satellite features confirm the Fe^3+^ state [27, 28, 29, 30]. The Co/Fe atomic ratios determined from XPS are NN = 2.96:1, NS = 2.98:1, and MP = 3.01:1, indicating a nearly identical Co/Fe stoichiometry across the three morphologies. Consistent with this, the Co 2p and Fe 2p spectra of NN, NS, and MP are almost completely overlapped in terms of peak position, peak shape, and satellite features. Additionally, no detectable Cl or S signals were observed in the high‐resolution spectra (Figure S1e,f), indicating that, within the detection limit of XPS, no appreciable CTAC‐ or SDBS‐derived Cl or S residues remain on the surfaces after synthesis and washing. Based on these XPS and XRD results, the three samples are considered to possess a highly consistent bulk crystal structure and surface chemical states. While the potential subtle effects of the surfactants used in synthesis on properties such as wettability or catalyst loading cannot be entirely ruled out, these characterizations provide a reasonable basis for attributing the observed electrochemical differences primarily to morphological factors. The detailed quantitative analysis of intrinsic activity is provided in Section 2.4 with C_dl_‐normalized LSV curves, which further supports that the performance variation originates mainly from morphology‐regulated bubble dynamics and mass transport rather than from differences in composition, crystal phase, or electrochemically active surface area.

Figure 2c–e show the SEM images of NN, NS, and MP. For NN, NN shows convex surfaces with uniform positive curvature. NS possesses planar walls with near‐zero curvature, while MP exhibits negative concave curvature. Here, the curvature used is not the mean or Gaussian curvature obtained from 3D surface reconstruction. Rather, we define an apparent 3D signed local profile curvature derived from the local solid–electrolyte interfacial contours extracted from SEM images. Briefly, a convex solid contour protruding toward the electrolyte is assigned a positive sign, a concave contour recessed into the electrode a negative sign, and a locally planar contour corresponds to a curvature close to zero. The sign of the curvature therefore matches the surface convexity of each structure, laying a geometric foundation for the subsequent bubble analysis. A detailed definition, the calculation procedure, and the full curvature distributions are provided in the Supporting Information and Figure S2.

#### 2.2.2 Pore Structure and Aerophobic Properties

As indicated by the SEM images, the three electrodes exhibit distinctly different morphologies, which are expected to give rise to substantially different interfacial mass‐transfer environments. To further elucidate how morphology governs electrode performance, mercury intrusion porosimetry and contact angle measurements were conducted to compare the pore structures and aerophobic characteristics of the three samples.

Figure S3 shows the pore size distributions of the three samples. Although the total pore volume of all electrodes is dominated by macropores (>10 µm), the NN sample exhibits a substantially higher fraction of pore throats below 0.5 µm than NS and MP. This hierarchical architecture, enriched with nanoscale pores, represents a key structural characteristic underlying the superior bubble behavior of NN. From a physical perspective, the high density of nanopores on the NN surface (2–50 nm and 50–500 nm) generates strong capillary pressure (*P_c_* ≈ 2γ/*r*).

Contact‐angle measurements were performed at room temperature using deionized water as the probe liquid to evaluate the wetting behavior of NN, NS, and MP (Figure 2f–k). NN absorbed a 1 µL droplet within 5 ms and NS absorbed a 1 µL droplet within 10 ms, indicating rapid wetting of both surfaces. By contrast, MP showed a contact angle of 129.88°, indicating considerably weaker liquid wettability. To directly characterize gas wettability at the solid‐liquid interface, underwater O_2_ bubble contact angles were measured (Figure S4). The apparent bubble contact angles, measured through the gas phase, were 146.07 ± 3.69° for NN, 142.32 ± 9.27° for NS, and 53.28 ± 7.23° for MP. Thus, NN and NS both exhibit strong and comparable underwater aerophobicity, with NN showing a slightly larger nominal value, whereas MP has a substantially greater affinity for bubble attachment.

Notably, the trends revealed by the pore‐structure analysis, water contact angles, and underwater O_2_ bubble contact angles do not follow an identical sample ranking, demonstrating that no single static descriptor can independently predict either dynamic bubble release or electrochemical performance. Mercury intrusion porosimetry reveals that only NN contains a substantially greater fraction of nanopores (2–50 nm and 50–500 nm). Because capillary liquid retention depends jointly on pore radius and liquid wettability, the fine‐pore architecture of NN enables its favorable wettability to more effectively promote electrolyte infiltration and sustain a liquid‐filled porous interface and interfacial liquid layer during continuous gas evolution.

In comparison, NS possesses a similarly aerophobic static wetting state but contains fewer nanopores, providing less structural assistance for capillary liquid retention and interfacial liquid replenishment. Nevertheless, its strong underwater aerophobicity is consistent with its slightly better bubble‐release dynamics than MP. Although MP has a pore‐size distribution broadly similar to that of NS, its much weaker liquid wettability and underwater aerophobicity result in a greater tendency toward bubble retention. Thus, the bubble‐release behavior follows the order NN > NS > MP, although the difference between NS and MP is relatively small. A detailed discussion of the bubble dynamics is presented in Section 2.3.

### 2.3 Bubble Dynamics Analysis

#### 2.3.1 In Situ Visualization of Bubble Detachment Diameter

The macroscopic electrochemical performance of an electrode, particularly its stability at high current densities, is governed by the combined effects of intrinsic catalytic activity, the density of accessible active sites, and mass transport. Together, these factors determine how efficiently the electrode operates under practical conditions. High‐speed in situ imaging of bubble detachment provides direct visual and quantitative evidence for elucidating performance differences among electrodes with different surface architectures.

**FIGURE 3:**
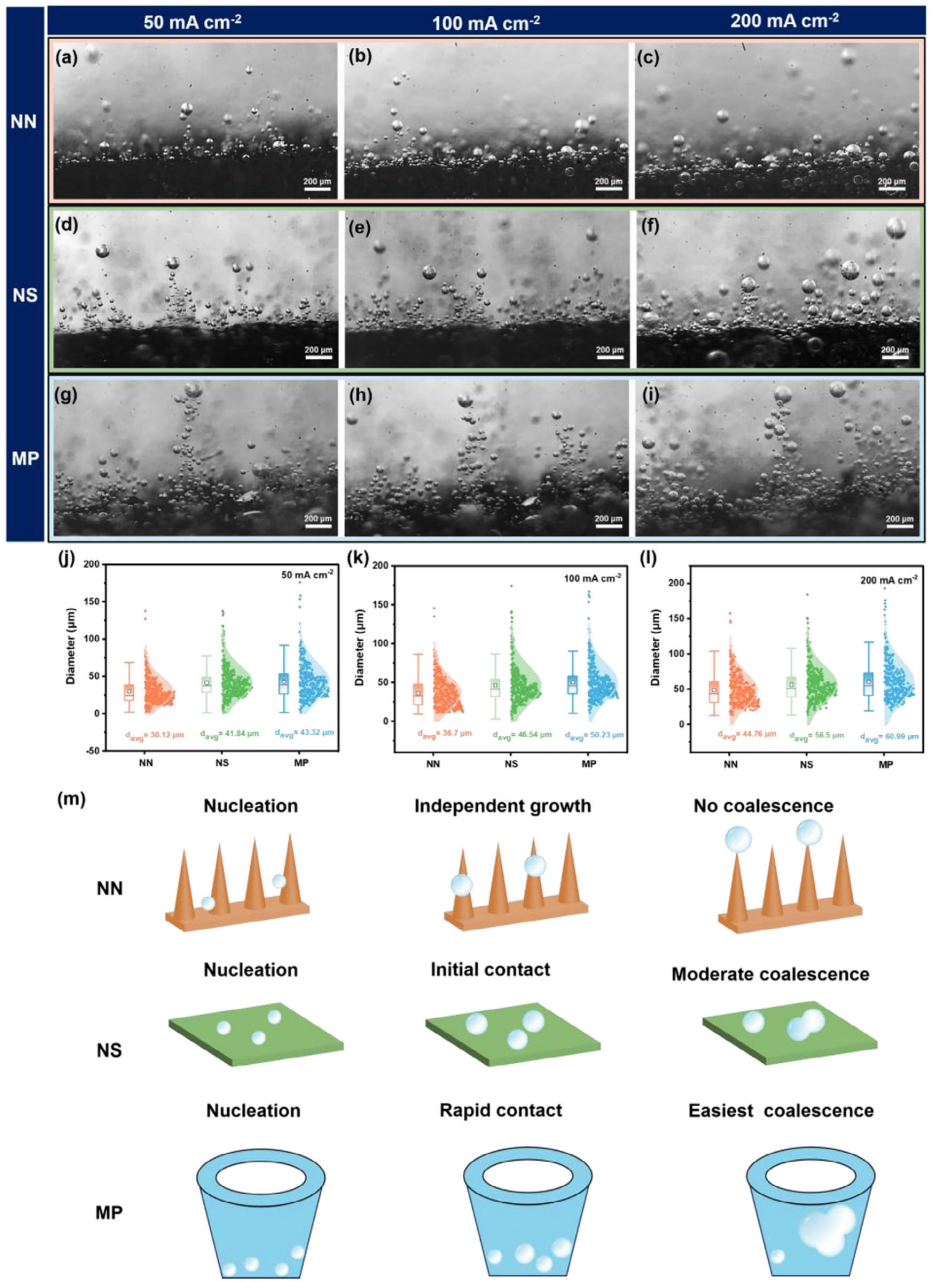
(a–c) Bubble evolution images on NN at current densities of 50, 100 and 200 mA cm^−2^, respectively. (d–f) Bubble evolution images on NS at 50, 100, and 200 mA cm^−2^, respectively. (g–i) Bubble evolution images on MP at 50, 100, and 200 mA cm^−2^, respectively. (j–l) Average bubble diameters of the three electrocatalysts as a function of applied current density. (m) Bubble coalescence evolution comparison among NN, NS and MP with different surface curvatures.

Bubble diameters were systematically quantified for the NN, NS, and MP electrodes at current densities of 50, 100, and 200 mA cm^−2^. Figure 3a–i show representative in situ images of bubbles on the three electrodes acquired at these current densities. We recorded several consecutive high‐speed video segments within a total observation period of 2 min. Frames were extracted from different temporal positions across the entire observation window using the same sampling procedure for all electrodes. A total of 300 bubble observations, pooled from the extracted frames and video segments, were analyzed for each electrode under each condition. As shown in Figure 3j–l, the average bubble detachment diameters for NN were 30.12, 36.7, and 44.76 µm at 50, 100, and 200 mA cm^−2^, respectively. The corresponding values for NS were 41.84, 46.54, and 56.5 µm, while those for MP were 43.32, 50.23, and 60.99 µm. The experimentally measured bubble diameters exhibited the same monotonic trend observed in the simulations.

At the same current density, NN consistently showed the smallest average bubble detachment diameter and the narrowest size distribution, indicating weaker bubble adhesion at the interface and faster bubble release. This behavior stems from the positive convex curvature of NN. The convex surface shortens bubble contact lines and weakens interfacial pinning, which facilitates bubble detachment [31, 32]. In contrast, MP with continuous gradient negative concave curvature enables extended bubble contact lines and strengthened interfacial pinning. This accounts for its larger bubble size, slower detachment kinetics, and more severe bubble retention, which are likely to impede mass transport and increase interfacial resistance. These observations are in good agreement with the simulation, MIP, and contact angle results discussed above.

#### 2.3.2 Fast Fourier Transform (FFT)

Fast Fourier transform (FFT), an efficient implementation of the discrete Fourier transform (DFT), was employed to convert the signal from the time domain into the frequency domain, thereby enabling the identification of frequency dependent features. In water electrolysis, FFT is primarily used to resolve the frequency characteristics of signal fluctuations, which can then be correlated with bubble dynamics, including nucleation, growth, and detachment, thus providing a quantitative basis for optimizing electrode structures and operating conditions [33, 34, 35]. Following the frequency analysis method reported by Kwan and co‐workers [36], selected frequency windows were examined to minimize the influence of the potentiostat and experimental background. In this work, chronopotentiometric measurements were performed for the three electrodes using a Biologic SP‐300 (Figure S5), with a sampling interval of 1.6 ms (625 Hz) over a total acquisition time of 30 s. After the system had stabilized for 25 s, the final 5 s of the voltage signal were extracted for FFT analysis. The voltage fluctuations were transformed into the frequency domain and divided into three frequency ranges, namely low frequency (0.03–1 Hz), medium frequency (1–10 Hz), and high frequency (10–50 Hz). The low‐frequency region corresponds to the slow evolution of large bubbles, whereas the medium‐ and high‐frequency regions are associated with the rapid nucleation, growth, and detachment of small bubbles.

**FIGURE 4:**
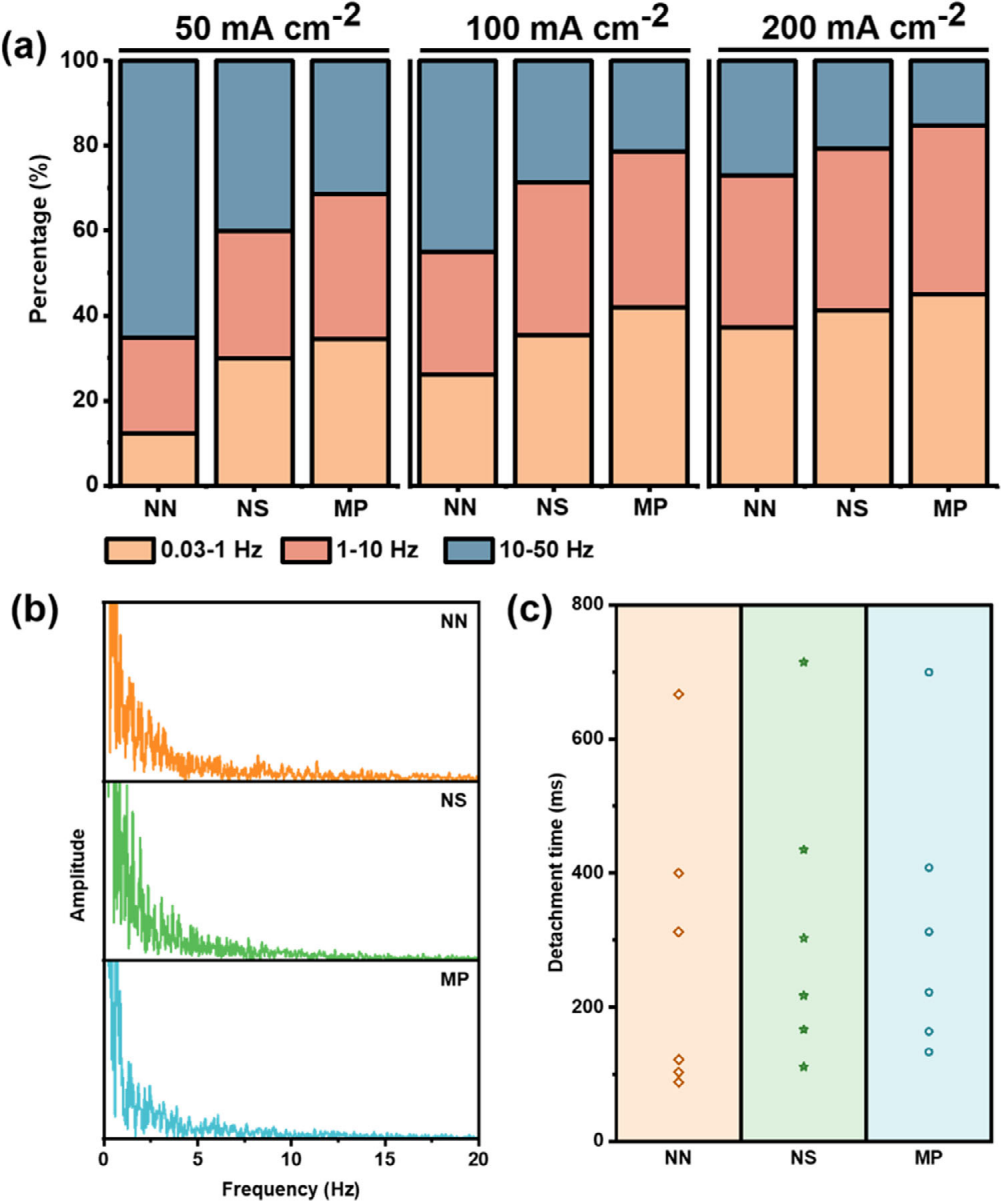
(a) FFT spectra of the voltage‐time signals of NN, NS, and MP under galvanostatic operation. (b) Amplitude‐frequency plots derived from the FFT results for each electrode. (c) Characteristic bubble detachment time for each electrode.

As shown in Figure 4a, the FFT spectra of the voltage fluctuations exhibit pronounced structure dependence across the three samples. Compared with NN, NS and MP show progressively higher signal fractions in the low‐frequency range (0.03–1 Hz), while their signal fractions in the medium‐frequency (1–10 Hz) and high‐frequency (10–50 Hz) ranges decrease accordingly. For example, at 50 mA cm^−2^, the low‐frequency fractions of NN, NS, and MP are 12.234%, 29.945%, and 34.411%, respectively, which indicates that bubble evolution on NN is more strongly governed by a high‐frequency detachment mode involving smaller bubbles. When the same sample is compared across different current densities, the low‐frequency fraction increases progressively as the current density increases, whereas the medium‐ and high‐frequency fractions decrease continuously, with the high‐frequency range typically showing the largest decline. The same trend is observed across all samples, indicating that bubble behavior changes systematically as the current density increases.

Based on the FFT results, amplitude‐frequency spectra were plotted (Figure 4b), from which the characteristic bubble frequency of each sample was obtained. The corresponding characteristic bubble detachment time was then derived using the reciprocal relation between time and frequency (*t* = 1/*f*), as shown in Figure 4c. In Figure 4b,c, NN exhibits the strongest high‐frequency response in the frequency domain, which directly reflects faster bubble detachment kinetics. By contrast, the high‐frequency components gradually diminish in NS and MP, indicating that bubble‐related mass‐transfer efficiency decreases as the surface morphology changes. On the time scale, the bubble detachment time shows clear interface dependence: NN exhibits the shortest detachment time, which is mainly concentrated around 100 ms, indicating that bubbles remain on the surface only briefly and that active sites can be rapidly re‐exposed. NS shows intermediate detachment times with a broader distribution, which suggests slightly longer bubble retention than NN. This result suggests that the sheet‐like morphology introduces local bubble‐pinning sites, which slightly prolong bubble retention and require bubbles to grow larger before detachment. MP exhibits an even longer bubble detachment time and shows almost no distribution within the 100 ms range, which indicates that prolonged bubble retention hinders mass transport at the reaction interface. Overall, from NN to MP, the bubble behavior evolves from fast, high‐frequency detachment to slower, low‐frequency detachment with increased surface retention.

### 2.4 Electrochemical Performance and Mass‐Transfer Characteristics

**FIGURE 5:**
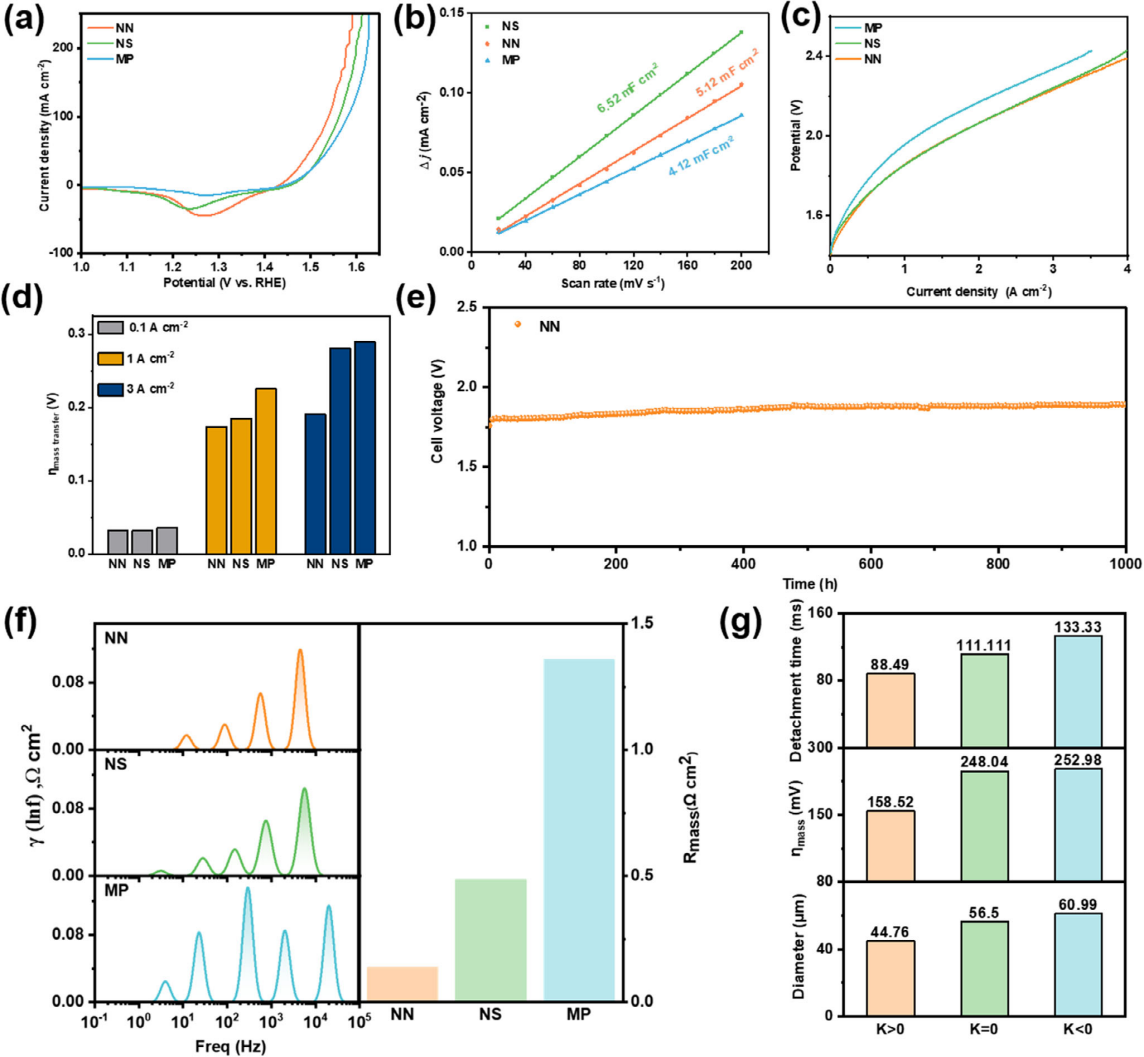
(a) LSV curves of NN, NS and MP. (b) C_dl_ of NN, NS and MP. (c) Polarization curves of NN, NS and MP operated in AEMWE;(d) The overvoltage of AEMWE was subdivided into η_*mass*_ at different current densities. (e) The durability voltage‐time plots for AEMWE using NN at 1 A cm^−2^ at 60°C. (f) DRT analysis of 1 A cm^−2^. (g) Bubble behavior and mass transport overpotential under different curvature.

To systematically evaluate the oxygen evolution reaction (OER) performance of electrodes with different surface morphologies, linear sweep voltammetry (LSV) was performed. As plotted in Figure 5a, NN delivers the best OER activity in alkaline electrolyte, requiring an overpotential of only 220 mV to reach 10 mA cm^−2^, which is lower than that of NS (247 mV) and MP (278 mV). The measured double‐layer capacitance (C_dl_), however, follows the order NS > NN > MP, as determined from the non faradaic CV measurements (Figures 5b and 1, S7). Because the material specific capacitance (C_s_) was not independently determined, an absolute ECSA was not calculated; instead, C_dl_ is used only as a relative proxy for the electrochemically accessible interfacial area.

To account for differences in accessible surface area, the LSV currents were further normalized by C_dl_ (Figure S6). Notably, NN retains higher C_dl_ ‐normalized OER activity than NS in the high current density regime, despite its lower C_dl_. Thus, the larger electrochemically accessible area indicated by the C_dl_ of NS does not translate into better active‐site utilization during vigorous oxygen evolution. Nevertheless, C_dl_ normalization provides only an approximate area‐based comparison, and the present correlations do not establish bubble mediated mass transport as the sole or dominant origin of the activity differences; contributions from other morphology‐dependent factors cannot be excluded.

To further validate the electrochemical trend in a practical testing configuration, polarization curves were measured in an anion exchange membrane water electrolyzer (AEMWE) (Figure 5c). The same activity order was obtained, which again confirms the superior catalytic performance of NN. Polarization data were used to separate the total overpotential into ohmic, kinetic, and mass‐transfer contributions (Figure S8). The mass‐transfer contribution, η_*mass*_, was then plotted against current density (Figure 5d). Although η_*m*_ increases with current density for all three electrodes, NN shows the smallest increase, which provides direct evidence of its more favorable mass‐transfer characteristics.

This advantage can be understood from the combined effects of surface structure and bubble behavior. The tip‐rich morphology of NN promotes rapid O_2_ bubble detachment, especially at high current densities. As a result, OH^−^ transport to active sites remains more continuous and efficient, which mitigates concentration polarization [37, 38]. In contrast, the larger and more persistent bubbles on NS and MP form stronger mass‐transfer barriers, causing η_*mass*_ to increase more sharply and the performance to deteriorate more rapidly in the high‐overpotential region. These results indicate that the needle‐like structure enables a better balance between catalytic activity and mass‐transfer efficiency.

The improved electrochemical performance of NN is further reflected in its stability. In a three electrode configuration, a 100 h galvanostatic test was conducted at 200 mA cm^−2^ (Figure S9). NN exhibits excellent stability with negligible potential decay, whereas NS and MP both show noticeable potential increases after prolonged operation, indicating activity loss. To assess device‐level durability, the NN electrode was assembled into a membrane electrode assembly (MEA) and tested in a water electrolyzer under galvanostatic operation for 1000 h (Figure 5e). At 1 A cm^−2^, the cell voltage increased from 1.76 to 1.88 V over 1000 h, demonstrating a low degradation rate and excellent operational stability. Electrochemical impedance spectroscopy (EIS) combined with distribution of relaxation times (DRT) analysis was used to decompose impedance losses in the water electrolysis system. EIS was tested at 0.1 Hz–100 kHz with an AC amplitude of 5% of the DC. Data validity was checked by Kramers‐Kronig transformation, and overlapping processes were deconvoluted by the model free DRT method. The DRT spectra were clearly divided into three characteristic regions: the low‐frequency region (< 40 Hz) corresponding to gas‐liquid mass transport, the medium‐frequency region (40 Hz–3 kHz) assigned to OER charge transfer, and the high‐frequency region (> 3 kHz) related to ion conduction. The impedance of each process was quantitatively calculated by integrating the peak area of each region. Figure 5f shows that positive‐curvature NN favored rapid bubble detachment without bubble stagnation, showing the lowest mass transport resistance and the smallest DRT low‐frequency mass transport peak. Near‐zero curvature NS exhibited moderate mass transport resistance. In contrast, negative‐curvature MP easily induced bubble accumulation and clogging, remarkably increasing mass transport resistance and enhancing the corresponding DRT low‐frequency peaks. These results indicate that the transition from positive to negative curvature aggravates gas‐liquid mass transport inhibition, and DRT can accurately distinguish mass transport differences induced by various morphologies. Figure 5g compares bubble detachment diameter, detachment time, and mass transport overpotential across positive, near‐zero, and negative curvature surfaces. With surface curvature varying from positive convex to near‐zero planar and then to negative concave, bubble detachment diameters and detachment time increase gradually, together with elevated mass transport overpotential. The convex positive curvature reduces bubble pinning at the interface and promotes bubble departure. Conversely, the concave negative curvature extends the solid‐liquid contact line and enhances bubble adhesion, resulting in larger bubbles, delayed detachment, and suppressed mass transport.

## 3 Conclusion

In summary, this work identifies electrode topological curvature as a key factor governing bubble dynamics and mass transport loss in OER. The NN electrode with positive convex curvature enables small bubble departure diameter and fast detachment, leading to the lowest mass transport overpotential. In contrast, the MP electrode with negative concave curvature causes serious bubble pinning and aggregation even with high porosity. These results confirm that curvature dominates bubble kinetics beyond porosity, which resolves the long ignored issue that high porosity does not guarantee efficient gas release. This work provides a universal curvature‐based design strategy for high performance OER electrocatalysts.

## Author Contributions

**Zhiqing Tang**: investigation, writing – original draft, visualization, writing – review and editing. **Ziyun Zhang**: software. **Huicun Gu**: software. **Baoxin Wu**: writing – review and editing. **Kejun Yan**: visualization, data curation. **Jiahui Luo**: methodology. **Yuting Jiang**: methodology, formal analysis. **Cailin Xiao**: methodology, validation. **Yu Xu**: methodology. **Lin Zeng**: funding acquisition, conceptualization, writing – review and editing, project administration, resources, supervision.

## Conflicts of Interest

The authors declare no conflicts of interest.

## Supporting information




**Supporting File**: advs77658‐sup‐0001‐SuppMat.docx.

## Data Availability

Research data are not shared.

## References

[1] C. Leggerini , M. Bannò , and M. Dal Molin , “Hydrogen Innovation: An Exploration of Its Determinants Across Europe,” Energy Policy 204 (2025): 114675, 10.1016/j.enpol.2025.114675.

[2] S. W. Boettcher , “Introduction to Green Hydrogen,” Chemical Reviews 124, no. 23 (2024): 13095–13098, 10.1021/acs.chemrev.4c00787. 39659176

[3] V. Khaligh , A. Ghezelbash , J. Liu , W. Won , J. Koo , and J. Na , “Multi‐period Hydrogen Supply Chain Planning for Advancing Hydrogen Transition Roadmaps,” Renewable and Sustainable Energy Reviews 200 (2024): 114536, 10.1016/j.rser.2024.114536.

[4] R. Iwata , L. Zhang , K. L. Wilke , et al., “Bubble Growth and Departure Modes on Wettable/Non‐wettable Porous Foams in Alkaline Water Splitting,” Joule 5, no. 4 (2021): 887–900, 10.1016/j.joule.2021.02.015.

[5] P. A. Kempler , R. H. Coridan , and L. Luo , “Gas Evolution in Water Electrolysis,” Chemical Reviews 124, no. 19 (2024): 10964–11007, 10.1021/acs.chemrev.4c00211. 39259040

[6] J. Pan , Z. Chen , P. Wang , et al., “The overall water splitting of CdS/Ti_3+_‐SrTiO_3_ core–shell heterojunction via OER enhancement of MnOx nanoparticles,” Chemical Engineering Journal 424 (2021): 130357, 10.1016/j.cej.2021.130357.

[7] C. Zhang , W. Xu , S. Li , et al., “Core‐shell Heterojunction Engineering of Ni_0.85_Se‐O/CN Electrocatalyst for Efficient OER,” Chemical Engineering Journal 454 (2023): 140291, 10.1016/j.cej.2022.140291.

[8] W. Zhang , X. He , J. Huang , and D. Chen , “Two‐phase Structure Matching Strategy Promotes Formation of Built‐in Electric Fields in Eu‐Co_3_O_4_/NiFe LDH Heterojunction for Anion Exchange Membrane Water Electrolysis,” Chemical Engineering Journal 527 (2026): 171696, 10.1016/j.cej.2025.171696.

[9] A. Huang , R. Xu , D. Zhao , X. Gao , and Z. Chen , “Self‐driven Coupling Field in Fe_3_O_4_@NiFe‐LDH/MnCO_3_ Heterojunction for Electrocatalytic Water Oxidation at High Current Densities,” Chemical Engineering Journal 521 (2025): 166883, 10.1016/j.cej.2025.166883.

[10] N. Zhang , Z. Song , X. Geng , et al., “Unlocking Efficient Water/Seawater Oxidation: The Dual‐role of Pt Dopants in Accelerating Surface Reconstruction and Switching Anodic Reaction Mechanisms,” Applied Catalysis B: Environment and Energy 385 (2026): 126331, 10.1016/j.apcatb.2025.126331.

[11] X. Li , J. Wang , H. Xue , et al., “Tuning α‐MnOOH Formation via Atomic‐Level Fe Introduction for Superior OER Performance,” Advanced Functional Materials 35, no. 34 (2025): 2503360, 10.1002/adfm.202503360.

[12] Y. Sun , J. Chen , L. Liu , H. Chi , and H. Han , “The Mechanism of OER Activity and Stability Enhancement in Acid by Atomically Doped Iridium in γ‐MnO_2_ ,” Chinese Journal of Catalysis 69 (2025): 99–110, 10.1016/s1872-2067(24)60201-9.

[13] J. Liu , J. Sun , Y. R. Hao , et al., “Oriented Assembly of Boundary‐Engineered Heterojunctions via Cerium Single‐Atom Doping for Enhanced Oxygen Evolution,” Advanced Functional Materials 35, no. 43 (2025): 11739, 10.1002/adfm.202511739.

[14] D. Yuan , X. Dou , Q. Zhang , et al., “Independent Tuning of Intermediate Energetics for Enhanced Oxygen Evolution via Synergistic Defects,” Advanced Functional Materials 35, no. 12 (2025): 2417098, 10.1002/adfm.202417098.

[15] W. Zhu , M. Ma , D. Gao , et al., “Establishing the Link between Oxygen Vacancy and Activity Enhancement in Acidic Water Oxidation of Trigonal Iridium Oxide,” Angewandte Chemie International Edition 64, no. 13 (2025): 202423353, 10.1002/anie.202423353. 39794300

[16] H. Zhou , J. Ban , Y. Shen , et al., “Strategies to Maximize the Oxygen Evolution Reaction in Layered Double Hydroxides by Electronic Defect Engineering,” eScience 5, no. 5 (2025): 100380, 10.1016/j.esci.2025.100380.

[17] X. Chen , T. Zhou , T. He , and Q. Liu , “Vacancy Engineering in the First Coordination Shell of Single‐Atom Catalysts for Enhanced Hydrogen and Oxygen Evolution Reactions,” Small 21, no. 14 (2025): 2412000, 10.1002/smll.202412000. 40059586

[18] L. Zhang , G. Li , H. Yan , et al., “Additive Manufacturing 3D Customizable Low‐cost Superwetting Polyacrylate‐based Hierarchically Micro‐nanoporous Lattice Anode for Energy‐saving Large‐current‐density Water Splitting Application,” Composites Part B: Engineering 245 (2022): 110189, 10.1016/j.compositesb.2022.110189.

[19] X. Chen , Y. Hou , J. Xu , et al., “0D‐2D Interaction: Pt Quantum Dots Trigger the Strain Effect of S‐NiFeP Nanosheet to Achieve Ampere‐level Current Density Overall Water Splitting Performance of Pt@S‐NiFeP/NF Electrode,” Applied Catalysis B: Environment and Energy 380 (2026): 125749, 10.1016/j.apcatb.2025.125749.

[20] Y. Yin , S. Wang , B. Chen , et al., “NiMoO(4)@CoFe‐LDH Anode Design to Improve Oxygen Bubble Transport in Dynamic Water Electrolysis,” Small 21, no. 45 (2025): 08103, 10.1002/smll.202508103. 40981379

[21] S. Pan , Y. Ye , C. Zhang , et al., “A Review of Designing Hierarchical Structure within Membrane Electrode Assembly for Water Electrolyzer,” Advanced Sciences (Weinh) 12, no. 36 (2025): 10546, 10.1002/advs.202510546. PMC12462931 40841861

[22] D. Guo , X. Guo , and X. Li , “Construction of 3D Porous MXene‐based Multiple Heterojunction Catalyst for Efficient Water Oxidation Reaction at High Current Density,” Journal of Colloid and Interface Science 691 (2025): 137441, 10.1016/j.jcis.2025.137441. 40163935

[23] Q. Lv , J. Han , X. Tan , W. Wang , L. Cao , and B. Dong , “Featherlike NiCoP Holey Nanoarrys for Efficient and Stable Seawater Splitting,” ACS Applied Energy Materials 2, no. 5 (2019): 3910–3917, 10.1021/acsaem.9b00599.

[24] H. Wang , X. Xue , M. Fan , et al., “Decoupling Bubble Nucleation From Catalysis to Boost Cu x O/NiO Electrocatalytic Water Splitting,” Nano Letters 26, no. 3 (2026): 1059–1067, 10.1021/acs.nanolett.5c04437. 41525175

[25] Y. Lai , L. Wang , S. Wang , et al., “Superhydrophilic/Superaerophobic Co_3_O_4_ Nanoneedle Array Electrocatalysts for Efficient Overall Water Splitting,” International Journal of Hydrogen Energy 178 (2025): 151704, 10.1016/j.ijhydene.2025.151704.

[26] Y. Chu , Z. Wang , X. Zhang , et al., “Ultra‐thin Nanohoneycomb Porous CoMoO_4_ With Excellent Catalytic Performance for Water Splitting at Large Current Densities,” Surfaces and Interfaces 44 (2024): 103737, 10.1016/j.surfin.2023.103737.

[27] M. C. Biesinger , B. P. Payne , A. P. Grosvenor , L. W. M. Lau , A. R. Gerson , and R. S. C. Smart , “Resolving Surface Chemical States in XPS Analysis of First Row Transition Metals, Oxides and Hydroxides: Cr, Mn, Fe, Co and Ni,” Applied Surface Science 257, no. 7 (2011): 2717–2730, 10.1016/j.apsusc.2010.10.051.

[28] D. Li , R. Xiang , F. Yu , et al., “In Situ Regulating Cobalt/Iron Oxide‐Oxyhydroxide Exchange by Dynamic Iron Incorporation for Robust Oxygen Evolution at Large Current Density,” Advanced Materials 36, no. 5 (2024): 2305685, 10.1002/adma.202305685. 37747155

[29] Y. Wang , Y. Zhang , S. Wang , et al., “Fe Coordination Environment Modulating Oxygen Evolution Reaction Properties of Cobalt Site,” Small 21, no. 26 (2025): 2500121, 10.1002/smll.202500121. 40370292

[30] S. Li , M. Zhou , H. Wu , et al., “High‐efficiency Degradation of Carbamazepine by the Synergistic Electro‐activation and Bimetals (FeCo@NC) Catalytic‐activation of Peroxymonosulfate,” Applied Catalysis B: Environmental 338 (2023): 123064, 10.1016/j.apcatb.2023.123064.

[31] B. Pang , Y. Liu , L. Shan , et al., “Surface Tip Effects Enhanced Performance of Proton Exchange Membrane Water Electrolyzers,” Nano Letters 25, no. 32 (2025): 12334–12342, 10.1021/acs.nanolett.5c02984. 40755435

[32] X. Zhang , S. Zhu , Y. Xu , and Y. Wang , “Tip Effect Assisted High Active Sites for Oxygen Evolution Reaction Tuned Using Transition Metals (Cr, Fe and Mo) Doped With CoP,” Journal of Materials Chemistry A 11, no. 43 (2023): 23270–23277, 10.1039/d3ta04723g.

[33] X. Wei , Y. Umehara , H. Nakajima , K. Ito , A. Etoh , and S. Mori , “Effect of Mesh Size of Ni Wire Mesh Electrodes on Alkaline Water Electrolysis Performance: A Study Based on the Observation of Bubble Departure Behavior,” International Journal of Hydrogen Energy 120 (2025): 189–200, 10.1016/j.ijhydene.2025.03.267.

[34] S. Yuan , C. Zhao , X. Mei , et al., “Bubble Management in PEM Water Electrolysis via Imprinting Patterned Grooves on Catalyst Layer,” International Journal of Heat and Mass Transfer 212 (2023): 124249, 10.1016/j.ijheatmasstransfer.2023.124249.

[35] S. Yuan , C. Zhao , L. Luo , et al., “Revealing the Role of the Ionomer at the Triple‐Phase Boundary in a Proton‐Exchange Membrane Water Electrolyzer,” The Journal of Physical Chemistry Letters 15, no. 19 (2024): 5223–5230, 10.1021/acs.jpclett.4c00851. 38717392

[36] J. T. H. Kwan , A. Nouri‐Khorasani , A. Bonakdarpour , D. G. McClement , G. Afonso , and D. P. Wilkinson , “Frequency Analysis of Water Electrolysis Current Fluctuations in a PEM Flow Cell: Insights Into Bubble Nucleation and Detachment,” Journal of The Electrochemical Society 169, no. 5 (2022): 054531, 10.1149/1945-7111/ac707f.

[37] S. Ding , Z. Li , G. Lin , L. Wang , A. Dong , and L. Sun , “Enhancing Mass Transfer in Anion Exchange Membrane Water Electrolysis by Overlaid Nickel Mesh Substrate,” ACS Energy Letters 9, no. 8 (2024): 3719–3726, 10.1021/acsenergylett.4c01568.

[38] L. Wan , J. Liu , Z. Xu , et al., “Construction of Integrated Electrodes With Transport Highways for Pure‐Water‐Fed Anion Exchange Membrane Water Electrolysis,” Small 18, no. 21 (2022): 2200380, 10.1002/smll.202200380. 35491509

