# Supplementary material for: Deciphering Structure‐Bubble‐Mass Transfer Relationships in Water Electrolysis Through Electrode Morphology Regulation
Source: Adv Sci (Weinh). 2026 Sep 13:e77658. Online ahead of print. doi: 10.1002/advs.77658 (PMC13572823; doi:10.1002/advs.77658)
Supplement: Supplementary file 1 [file ADVS-9999-e77658-s001.docx]

**Deciphering structure-bubble-mass transfer relationships in water electrolysis through electrode morphology regulation**

Zhiqing Tang^a,b^, Ziyun Zhang^a,b^, Huicun Gu^a,b^, Baoxin Wu^a,b,c^, Kejun Yan^a,b^, Jiahui Luo^a,b^, Yuting Jiang^a,b^, Cailin Xiao^a,b^, Yu Xu^a,b^, Lin Zeng^a,b,^*

^a^ Shenzhen Key Laboratory of Hydrogen Energy, Department of Mechanical and Energy Engineering, Southern University of Science and Technology, Shenzhen 518055, P. R. China.

^b^ SUSTech Energy Institute for Carbon Neutrality, Southern University of Science and Technology, Shenzhen 518055, P. R. China.

^c^ Department of Mechanical Engineering, The Hong Kong Polytechnic University, Hung Hom, Kowloon, Hong Kong SAR, P. R. China

***Corresponding authors:**

Lin Zeng (zengl3@sustech.edu.cn)

**Experimental section**

**Materials**

Raw materials of Iron nitrate nonahydrate (Fe(No_3_)_3_ 9H_2_O), Cobalt(II) nitrate hexahydrate Co(NO_3_)_2_·6H_2_O, urea, ammonium fluoride (NH_4_F), cetyltrimethylammonium chloride (CTAC), sodium dodecylbenzene sulfonate (SDBS), potassium hydroxide (KOH), was bought from Aladdin Industrial Corporation and used without further purification. Nafion and Versogen-A40 membrane for electrochemical tests were bought from Fuel cell store.

**Preparation of CoFe-LDH**

Nickel foam (NF) (1 cm × 4 cm) was chosen as the substrate for this study. The NF was pretreated by ultrasonication in a mixture of acetone and anhydrous ethanol (1:1 volume ratio) for 10 minutes, followed by ultrasonication in 3.0 M HCl for 30 minutes. Afterward, the NF was cleaned with distilled water three times and then immersed in distilled water for further use.

In the process of preparing NN, 582.06 mg of Co(NO_3_)_2_·6H_2_O, 202 mg of Fe(No_3_)_3_ 9H_2_O, 296 mg of NH_4_F and 600 mg of urea were dissolved in 65 mL of deionized (DI) water and stirred to form a homogeneous solution. The treated NF was then immersed in this solution and transferred to a 100 mL Teflon-lined stainless-steel autoclave. The autoclave was heated to 120 ℃ for 6 hours. After the reaction, the CoFe-LDH (NN) electrode was removed, rinsed with distilled water, and dried in ambient air. NS and MP were prepared by adding CTAC and SDBS respectively on this basis.

**Materials characterizations**

The morphology of the electrodes was analyzed using scanning electron microscopy (SEM, Sigma 300). X-ray diffraction (XRD, Bruker Advance D8) measurements were performed with Cu Ka radiation (λ=0.15416 nm). X-ray photoelectron spectra (XPS, Escalab 250Xi) were acquired on a Thermo SCIENTIFIC ESCALAB 250Xi system, using Al Kα (hυ = 1486.8 eV) as the excitation source.

**Contact angle**

The underwater bubble contact angles were measured using the captive-bubble method. For each electrode, measurements were performed at three spatially distinct locations, and the quantitative results are now reported as the mean ± standard deviation (n = 3). he bubble contact angle, $\theta_{B}$, was defined as the angle measured through the gas phase, i.e., the angle between the tangent to the bubble profile at the three-phase contact line and the solid surface on the bubble side. Under this convention, a larger $\theta_{B}$ indicates lower underwater bubble affinity and stronger underwater aerophobicity.

**Electrochemical measurements**

Electrochemical tests were performed using an Ivium Stat and Solartron electrochemical workstation with a three-electrode system at room temperature (~25 ℃). The as-prepared electrodes were used as the working electrode, a graphite rod served as the counter electrode, and an Hg/HgO electrode was used as the reference electrode. The electrolyte was 1.0 M KOH (pH=13.9). Potentials versus Hg/HgO were converted to the reversible hydrogen electrode (RHE) scale using the equation $E_{\mathrm{RHE}}=E_{Hg/HgO}+0.098+0.059\times pH$. The OER overpotentials were calculated using: $\eta=E_{\mathrm{RHE}}-1.23 V.$Polarization curves for OER were recorded by linear sweep voltammetry at a scan rate of 5 mV s^-1^ with 85% iR compensation. Tafel slopes were determined by plotting overpotential with 85% iR compensation versus log(current density).

The electrochemical double-layer capacitance (C_dl_) was assessed by measuring scan-rate dependent CV curves within the potential range free of Faradaic currents. The C_dl_-normalized current was calculated as $j_{C_{dl}}=\frac{j_{geo}}{C_{dl}}$ Because was not determined, was not converted into an absolute ECSA. Long-term stability was evaluated by chronopotentiometry at constant current densities.

**AEM water electrolyzer setup**

The AEM water electrolyzer consists of the anode, cathode, gas diffusion layer, and anion exchange membrane (Versogen-A40). For comparison, commercial Pt/C powder (0.5 mg cm-2) was sprayed onto carbon paper to serve as the cathode electrocatalyst. The prepared NN, NS and MP were directly used as the anode to assemble the membrane electrode. Prior to use, the anion exchange membrane was soaked in 1 M KOH for over 24 hours. The active working electrode area was set to 1*1 cm^2^ in the single-cell configuration. Alkaline electrolytes were circulated only in the anode side using a pump from the electrolyte reservoir. The electrolyzer was heated by an integrated heating element, with temperature controlled by an automatic device. After stabilization, the performance of the cell was analyzed. All electrochemical tests were conducted on the single-cell, with polarization curves and long-term stability evaluated at 1000 mA cm^-2^ using the Land battery testing system, without iR compensation.

Overpotential Analysis

The cell voltage $E_{cell}$ is composed of the sum of the reversible cell potential $E_{rev}$ and the three main overpotentials $\eta_{i}$

$E_{cell} = E_{rev} + \eta_{kin} + \eta_{\Omega} + \eta_{m}$	(1)

Where $\eta_{kin}$ is the kinetic, $\eta_{\Omega}$ is the ohmic, and $\eta_{m}$ is the mass transport overpotential. At a temperature of 60 °C, the temperature dependent standard reversible potential, Erev, could be obtained from the literature:

$Erev = 1.5184 - 1.5421 \times{10}^{-3}T+9.523 \times{10}^{-5}TlnT + 9.84 \times{10}^{-8}T^{2}$(2)

Under current electrolyzer testing conditions, the $Erev$ was calculated to be 1.229 V,

EIS was used to measure the high frequency resistance (HFR) representing the total electronic cell resistance Rtot. The ohmic overpotential, $\eta_{\Omega}$, is therefore determined as:

$\eta\Omega= i \times Rtot = i \times HFR$	(3)

The kinetic overpotential was extracted using a Tafel model, in which the Tafel slope b and exchange current density i0 were the governing kinetic parameters. The Tafel model was fitted to iR-free AEMWE cell voltages between 4 and 100 mA cm^-2^. Assuming a non-polarizable OER, the entire kinetic overpotential of the cell was governed by OER with the Tafel slope b：

$\eta kin = b \times log (i/i0)$	(4)

Mass transport was defined as a sum of gaseous/liquid transfer in the porous transport layer/catalyst layer and ionic transport in the catalyst layers. In this study, it was calculated by subtracting the reversible cell potential and kinetic and ohmic overpotentials from the measured cell potential.

In situ visualization of bubble detachment diameter

The bubble behavior during gas evolution was directly observed and recorded using a Thousand Eyes Wolf G20C-Pro high-speed camera, which was positioned perpendicular to the electrode surface to capture the dynamic growth and detachment process of bubbles in real time. Images and videos were acquired at a sufficiently high frame rate to accurately identify the moment when bubbles depart from the catalyst surface. Subsequently, ImageJ software was employed to quantitatively analyze the bubble detachment diameter and number density. We recorded several consecutive high-speed video segments within a total observation period of 2 min. Frames were extracted from different temporal positions across the entire observation window using the same sampling procedure for all electrodes. A total of 300 bubble observations, pooled from the extracted frames and video segments, were analyzed for each electrode under each condition.

**LBM Simulation**

Previous studies have shown that the surface morphology of the catalyst layer alters the bubble contact area and the pinning strength, which in turn affect the bubble detachment diameter, detachment time, and surface coverage behavior. Building on this understanding, the complex electrochemical gas evolution process is simplified in this work by imposing a local gas source term near the solid-liquid interface, allowing bubbles to nucleate, grow, and detach on surfaces with different morphology. This simplification aims to first exclude the complexities introduced by full electrochemical coupling, thereby focusing on the intrinsic role of the geometric structure itself in bubble dynamics.

1. Numerical Model and Computational Domain

A three-dimensional D3Q19 color-gradient multiphase LBM was employed to describe the dynamic evolution of the gas-liquid interface. Interface stability was maintained through the collision, perturbation, and recoloring steps, while a body-force term was included to represent bubble migration and detachment under the combined effects of buoyancy and interfacial tension. The same discrete-velocity model, initial phase field, and numerical solution procedure were used for all three structures so that the comparison primarily reflected differences in structural morphology.

The computational domain was discretized into 100 × 100 × 200 lattice nodes with a lattice spacing of 0.04 μm, corresponding to a physical domain of 4 × 4 × 8 μm^3^. The needle, sheet, and mesh structures were embedded separately in domains of identical size. The initial fluid state, total gas-generation input, time-integration procedure, output interval, and event-identification criteria were kept consistent among the three cases, thereby establishing a unified basis for morphological comparison.

2. Unified Numerical Parameters and Boundary Conditions

The same relaxation time, lattice surface-tension parameter, recoloring parameter, body-force term, contact angle, and total gas-source strength were applied to all three structures. No parameter was individually adjusted for a specific morphology. The principal numerical settings are summarized in Table S1.

Table S1 Unified numerical parameters used in the LBM simulations

| Parameter | Symbol | Value |
| --- | --- | --- |
| Computational domain | N_x_ × N_y_ × N_z_ | 100 × 100 × 200 |
| Lattice spacing | Δx | 0.04 μm |
| Relaxation time | τ | 1.0 |
| Lattice surface-tension parameter | σ_LB_ | 0.075 |
| Recoloring parameter | β | 0.8 |
| Lattice body-force term | g_LB_ | 3.0 × 10⁻⁴ |
| Unified total gas source | Q_g,LB_ | 3.0 × 10^-2^ / step |

A bounce-back boundary condition was imposed on solid surfaces to enforce the no-slip condition. Periodic boundary conditions were applied in the x and y directions. An open pressure boundary, combined with non-equilibrium extrapolation, was prescribed at the top of the computational domain to allow detached bubbles to leave the domain while minimizing boundary-reflection effects on bubble dynamics.

3. Gas-Generation Treatment and Source Conservation

An equivalent gas mass source was used to represent gas generation. The source provided the same total gas-generation driving force in all three structures, enabling the effects of morphology on bubble nucleation, growth, coalescence, and detachment to be isolated without explicitly resolving the complete electrochemical reaction kinetics.

At each lattice time step, the prescribed total injected mass was first determined and all currently eligible source nodes were identified. The target mass was then distributed among these nodes using normalized weights. When the local storage capacity of a source node changed, the remaining mass was redistributed among the other eligible nodes. This procedure prevented differences in the number of effective source nodes among the structures from introducing deviations in the actual total gas-generation rate.

The source error was defined as $\varepsilon_{Q}=\frac{|M_{\mathrm{actual}} - M_{\mathrm{target}}|}{M_{\mathrm{target}}}$. Throughout the calculations, the cumulative actual injection remained in close agreement with the cumulative target injection, and both the single-step and cumulative errors were far below 1%. Therefore, the differences in detachment time and detachment diameter among the structures were not caused by inconsistent total gas input, but primarily reflected the effects of the structures on local gas accumulation, bubble coalescence, and contact-line pinning.

4. Bubble Identification, Lineage Tracking, and Detachment Criteria

Bubble identification was based on three-dimensional connected-component analysis. At each analysis time, individual gas-phase regions were labeled and their volume, equivalent diameter, centroid position, minimum height, and number of solid-contact voxels were extracted. The equivalent bubble diameter was calculated as $D_{\mathrm{eq}}={(\frac{6V}{\Pi})}^{\frac{1}{3}}$, where V is the three-dimensional volume of the corresponding bubble.

Figure S1. XPS spectra of NN, NS, and MP: (a) survey spectra; (b) high-resolution O 1s spectra; (c) high-resolution Co 2p spectra; and (d) high-resolution Fe 2p spectra. (e) high-resolution S 2p spectra. (f) high-resolution Cl 2p spectra.

Figure S2. Local curvature distribution of (a) MP, (b) NS and (c) NN.

Figure S3. (a-c) Pore volume of NN, NS, MP, and nickel foam; (d) pore size distribution of NN, NS, MP and nickel foam measured by mercury intrusion porosimetry.

Table S2. Mercury intrusion porosimetry results for NN, NS, MP, and nickel foam.

| Type | NN | NS | MP | Ni foam |
| --- | --- | --- | --- | --- |
| Porosity, % | 25.1464 | 32.4303 | 36.1089 | 40.4818 |
| Average pore diameter, nm | 39.45 | 73.83 | 66.22 | 159.53 |
| Volume density, g/mL | 1.7736 | 1.5487 | 1.7685 | 1.5807 |
| Median pore diameter (volume), nm | 29,564.34 | 36,357.45 | 39,402.92 | 31,102.43 |
| Median pore diameter (area), nm | 2.3695 | 8.78 | 8.85 | 15.59 |
| Total pore area, $m^{2}/g$ | 14.374 | 11.345 | 12.334 | 6.421 |

Figure S4. Bubble contact angels of (a) NN, (b) NS and (c) MP

Figure S5. Overpotential-time data for FFT analysis at different current densities.

Figure S6 C_dl_-normalized LSV curves of NN, NS and MP

Figure S7. CV curves recorded at scan rates from 20 to 200 mV s^-1^: (a) NS, (b) NN, and (c) MP.

Figure S8 Overpotential contributions to the polarization curves of the substrates (a) NN. (b)NS. (c) MP

Figure S9. Stability test at a current density of 200 mA cm^-2^.
